# Congenital hypothyroidism in two children affected by Sotos syndrome: a simple association?

**DOI:** 10.1186/s13052-025-02114-4

**Published:** 2025-09-29

**Authors:** Paolo Cavarzere, Stefania Munari, Marta Arrigoni, Vincenzo Raitano, Elena Fiorini, Alessandra Guzzo, Rossella Gaudino, Franco Antoniazzi

**Affiliations:** 1 Pediatric Division, Department of Pediatrics, University Hospital of Verona, Piazzale Stefani 1, Verona, 37126 Italy; 2 Child Neuropsychiatry Unit, University Hospital of Verona, Verona, Italy; 3 Laboratory Unit, Department of Neurosciences, Biomedicine and Movement Sciences, University of Verona, Verona, Italy; 4 Pediatric Clinic, Department Surgical Sciences, Dentistry, Gynecology and Pediatrics, University of Verona, Verona, Italy; 5 Regional Center for the Diagnosis and Treatment of Children and Adolescents Rare Skeletal Disorders. Pediatric Clinic, Department of Surgical Sciences, Dentistry, Gynecology and Pediatrics, University of Verona, Verona, Italy

**Keywords:** Congenital hypothyroidism, Sotos syndrome, Overgrowth, Cognitive delay, Case report

## Abstract

**Background:**

Congenital hypothyroidism (CH) is the most common congenital endocrine disorder and one of the most preventable causes of intellectual disability. The underlying etiology of CH can be thyroid dysgenesis or dyshormonogenesis, and in rare cases, CH can occur as part of a genetic syndrome. Sotos syndrome is a rare overgrowth disorder caused by pathogenic variants in the *NSD1* gene, characterized by excessive growth in infancy, distinctive facial features, and developmental delay.

**Case presentation:**

We describe two unrelated children with permanent CH and genetically confirmed Sotos syndrome. Both children were referred to our Pediatric Endocrinology Centre due to abnormal thyroid-stimulating hormone (TSH) values detected through neonatal screening. A permanent CH was confirmed in both cases: one patient had thyroid hypoplasia with the presence of only the right thyroidal lobe; the other one had an in-situ thyroid gland. The diagnosis of Sotos syndrome was made later in infancy. In the first case, auxological parameters at birth were within normal ranges and overgrowth became apparent after six months of age; in the second case, overgrowth was already manifest at birth, but the diagnosis was guided primarily by the neurodevelopmental delay.

**Conclusion:**

We describe two cases in which CH occurred with Sotos syndrome, and we hypothesize that this association may not be coincidental. To our knowledge, these are among the few reported cases of genetically confirmed Sotos syndrome associated with permanent congenital hypothyroidism. Further studies are needed to determine whether CH is a clinical feature of Sotos syndrome or an unrelated finding. We recommend early thyroid function testing in patients with Sotos syndrome and suggest suspecting Sotos syndrome in children presenting with CH, cognitive delay and overgrowth or additional congenital anomalies.

## Background

Congenital Hypothyroidism (CH) is an endocrine disorder characterized by a thyroid hormone (TH) deficiency at birth, and is one of the most preventable causes of intellectual disability [1, 2]. The incidence of CH lies between 1 in 2,000 and 1 in 3,000 newborns and has been progressively increasing over time [3]. For over 40 years, CH has been diagnosed through neonatal screening programs, at least in developed countries [4–7].

The most remarkable clinical features of CH include a wide posterior fontanel, neonatal hypotonia, persistent jaundice and poor feeding [4]. In addition, CH is associated with an increased risk of congenital malformations, mainly congenital heart defects. Other findings may be spiky hair, cleft palate, neurological abnormalities, and genitourinary malformations [8, 9].

CH is classified as transient or permanent. Transient CH refers to a temporary deficiency of TH production, with recovery of normal thyroid function within months or years. It is typically due to iodine deficiency or excess, but can also result from maternal antithyroid drugs consumption or inhibitory antibodies, or can result from genetic variants, such as those involving *DUOX* gene [10–12]. In contrast, permanent CH refers to a persistent deficiency of TH production requiring lifelong treatment. The primary causes of permanent CH include thyroid dysgenesis and dyshormonogenesis. Thyroid dysgenesis accounts for approximately 65% of CH cases, and is sometimes due to mutations in genes involved in thyroid development, such as *TSHR, PAX8, NKX2.1, FOXE1, NKX 2.5, GLIS3, JAG1, NTN 1, BOREALIN* and others [3, 13–17]. Variants in some of these genes have been associated with extrathyroidal manifestations, such as renal hemiagenesis (*PAX8*) [18], choreoathetosis (*NKX2.1)* [19], choanal atresia and cleft palate (*FOXE1*), congenital heart defects (*NKX2.5, JAG1)*, neonatal diabetes (*GLIS3*), and arthrogryposis (*NTN1*) [20].

The remaining 35% of cases are attributed to dyshormonogenesis, resulting from variants in *TPO, TG, DOUX1, DOUX2, DOUXA2, PDS*, and *SLC5A5* genes [3, 16, 17, 20–22]. CH associated with *PDS* variants can be characterized by a variable degree of sensorineural hearing loss.

CH is also commonly present in syndromic conditions such as Down syndrome [23–25], DiGeorge syndrome [26], Williams syndrome [27], and the rarer Kabuki syndrome [20, 28]. To date, no proven association between CH and Sotos syndrome has been reported. Here, we report two cases of children with Sotos syndrome who presented with permanent CH.

## Cases presentation

A six-day-old newborn female was referred to our Pediatric Endocrinology Centre due to an abnormal TSH level detected during newborn screening. She was born at 38 weeks of gestational age by C-section because of breech presentation. Her birth weight was 3,185 g (0.31 SDS), birth length 51 cm (1.3 SDS) and head circumference 36 cm (2.11 SDS). Parents were not related, and familial history was unremarkable.

The pregnancy was unplanned and characterized by periconceptional vaccination for measles, mumps and rubella. An amniocentesis carried out by maternal choice for fear that the previous vaccination could have caused problems to the fetus, ruling out any major chromosomal alteration. No non-invasive genetic screening was performed. Ultrasound during the third trimester revealed bilateral hydronephrosis and oligohydramnios. At birth, she was admitted to our Centre’s Neonatal Intensive Care Unit (NICU) because she needed mild ventilatory support, as consequence of an episode of apnea, cyanosis and hypotonia occurred on her third day of life. On that occasion, blood tests were performed excluding a neonatal sepsis and a research for rubella in urine that ruled out any infection. A neuropsychiatric evaluation, including electroencephalogram and cranial ultrasound, was normal; clinically, only cervical hypotonia was noted. Abdominal ultrasound confirmed left-sided hydronephrosis (6 mm). Given the screening result, we assessed thyroid function: TSH was 273 mIU/L (normal range 0.30–4.20), fT4 was 9 pmol/L (normal range 11.0–22.0) with thyroglobulin of 118.81 ng/mL. Tests for anti-thyroid antibodies (TG, TPO, TSHR) were negative.

Thyroid ultrasound identified only the right thyroid lobe, which appeared normal in structure and without focal lesions. The left lobe and isthmus were not visualized. We performed a ™−99-pertechnetate scintigraphy that documented a slight isotope uptake, located cranially compared to the normal anatomic position. Therefore, she was started a treatment on levothyroxine at the dosage of 9.5 mcg/kg/day.

In the first months of life, the patient experienced frequent illnesses: she was admitted to the hospital due to a late-onset neonatal sepsis and later for an episode of bronchiolitis that required a non-invasive ventilatory support with high flow nasal cannulas. At 3 months of age, she was admitted to the Pediatric Intensive Care Unit because of a hypovolemic shock with extreme lethargy following diarrhea and received intravenous rehydration. Rotavirus was detected on a sample of stools, but the attempt to feed her with formulated milk led to persistent vomiting and diarrhea, once again with metabolic acidosis. Following this episode, she was diagnosed with food protein induced enterocolitis syndrome (FPIES). She later developed two episodes of pyelonephritis caused by multidrug-resistant Klebsiella pneumoniae and Escherichia coli. We therefore performed more tests that ruled out an immunodeficiency and allowed us to detect a mild congenital cardiopathy due to an interatrial defect with left-to-right shunt and signs of enlargement of the right ventricle.

**Fig. 1:**
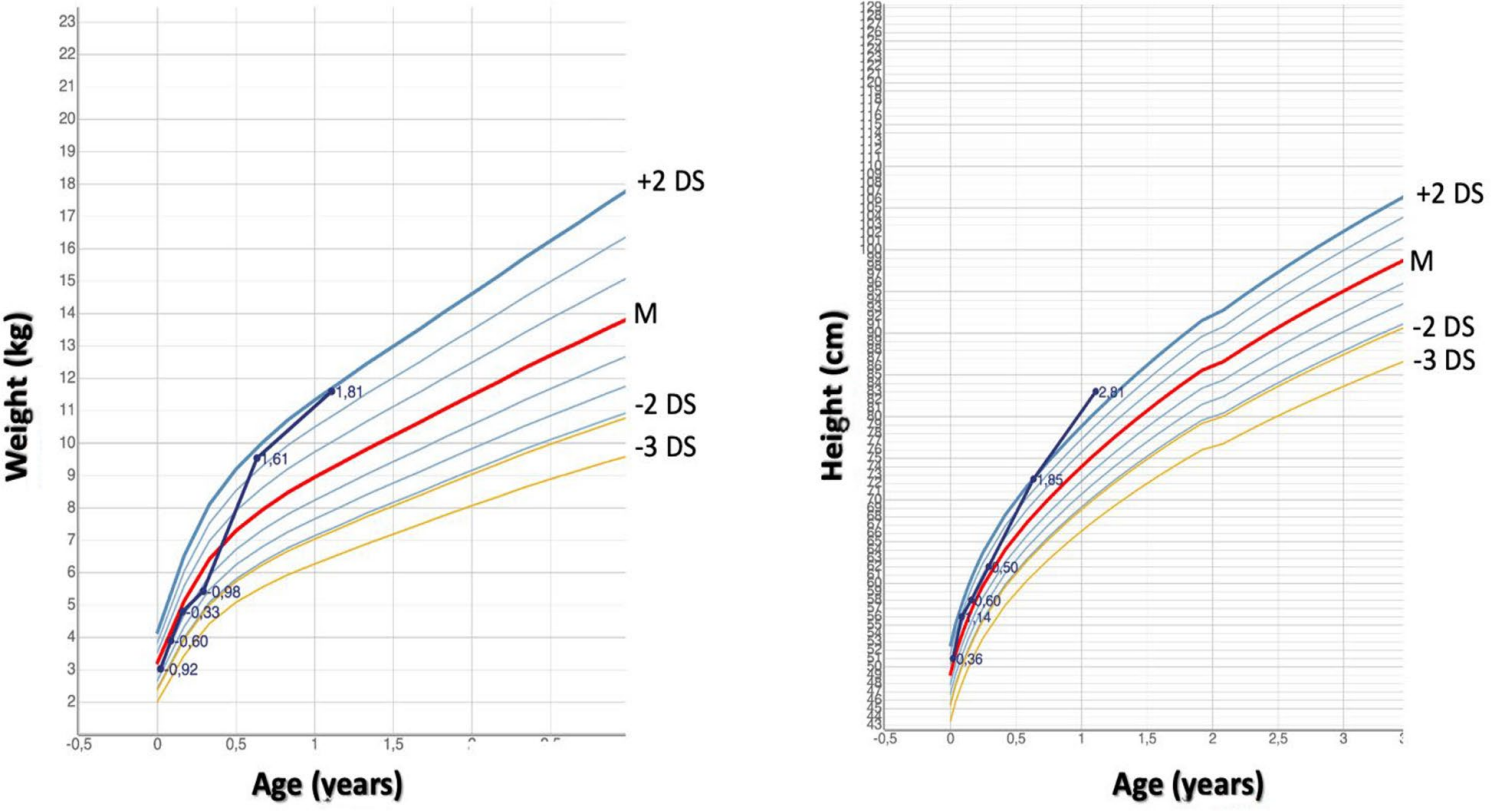
Growth charts for weight and height of patient 1 (according to Italian growth charts [29])

During the endocrinological follow-up, we observed progressive intellectual disability and overgrowth (Fig. 1). Given the presence of CH, mild congenital cardiopathy, hydronephrosis, psychomotor retardation and overgrowth we suspected a genetic cause. Comprehensive gene panel analysis identified a de novo mutation in the *NSD1* gene (c.3439G > T, p.(Glu1147*)), confirming a diagnosis of Sotos syndrome.

A 5-year old boy was referred to our Pediatric Endocrinology Centre soon after birth due to a positive newborn screening for CH.

He was born at term from a pregnancy characterized by maternal pre-eclampsia, risk of preterm birth at 32 weeks of gestational age, evidence of triventricular hydrocephalus at the fetal ultrasonography and magnetic resonance imaging and right pyelectasis. His parents were related: particularly the paternal grandfather was the maternal great-grandfather’s brother.

At birth, he presented a major cervical hypotonia and a noticeable ligamentous laxity, with scarce newborn reflexes and poor spontaneous motricity. No typical dysmorphic facial features were present. His anthropometric measures at birth were: birthweight 3,855 g (1.19 SDS), length 58 cm (5.47 SDS) and cranic circumference 37 cm (2.16 SDS).

He was admitted to the NICU of our hospital to monitor his neurological state and, secondarily, to manage frequent episodes of apnea with low oxygen saturation, which needed a non-invasive ventilatory support with high flow nasal cannulas. During his hospital stay, he underwent several neuropsychiatric evaluations with neuroimaging, which showed foci of intracranial hemorrhages despite normal blood clotting with subsequent encephalomalacia and a global delay in neurodevelopment concerning even his acoustic and visual performances. Moreover, he developed symptomatic seizures, which were successfully treated with phenobarbital. He underwent further analysis, such as a pneumological evaluation and a fibrolaringoscopy, which led to the diagnosis of vocal cords malacia; a polysomnography documented a severe obstructive dysfunction. Moreover, he had a postnatal Cytomegalovirus infection serologically documented, he developed a cholestasis with evidence of dysmorphic gallbladder and the echocardiography showed an atrial septal defect and a patent ductus arteriosus, which healed spontaneously.

For what concerns the endocrinological field, when recalled from the newborn screening his thyroid function was altered, showing a subclinical hypothyroidism with a TSH of 14.67 mIU/L (normal values 0.30–4.20 mIU/L) and normal fT3 and fT4, with negative anti-thyroperoxidase and anti-thyroglobulin antibodies. The thyroid ultrasound evidenced a thyroid in situ with an inhomogeneous aspect; a substitutive levothyroxine therapy was begun at the age of 15 days. The levothyroxine dosage underwent a progressive titration until the normalization of the hormonal panel; the patient was finally discharged from the hospital at the age of three months, with a close and multidisciplinary follow-up scheduled.

**Fig. 2:**
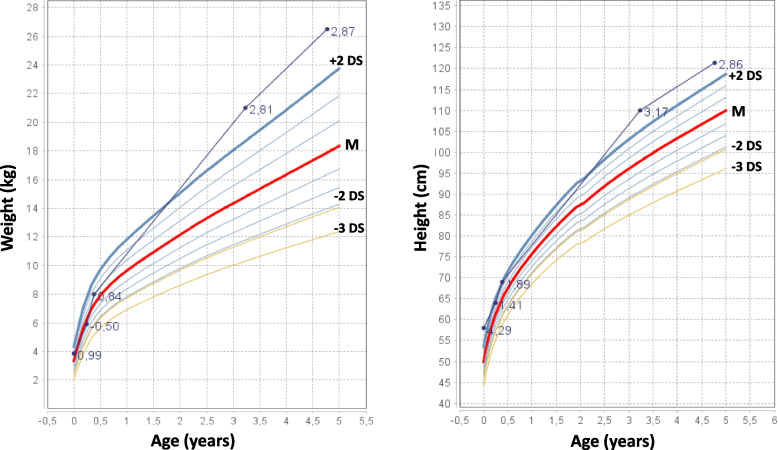
Growth charts for weight and height of patient 2 (according to Italian growth charts [29])

His follow-up was complicated by the Sars-CoV2 pandemics and by the lack of consistency in the endocrinological evaluations, as his parents missed several planned visits. Despite such a difficult and inconsistent follow-up, the anthropometric evaluations throughout the years have always evidenced a stature above the 97th centile (Fig. 2).

At the last neurological visit, he appeared in good general conditions despite he had no autonomy in sphincter control, in eating and in dressing himself. He doesn't speak, emits vocalizations but he seems to understand simple commands.

The association between the neurodevelopmental delay, hypotonia, ligamentous laxity, seizures and high stature led to a strong suspect for a genetic syndrome. A broad NGS panel of genes related to epilepsy and intellectual disability was performed, which resulted positive for a de novo heterozygous mutation of the gene *NSD1* (NM_022455.5:C.2954_2955delCT, p.Ser985Cysfs*25) responsible for Sotos syndrome.

## Discussion and conclusions

Our manuscript describes two children affected by Sotos syndrome who presented with CH, leading us to hypothesize a potential correlation between this syndromic condition and CH. The clinical, auxological, and biochemical characteristics of the two patients are summarized in Table 1.

**Table 1:** Clinical, auxological, and biochemical characteristics of the two patients

	Patient 1	Patient 2
Sex	Female	Male
Ethnicity	Indian	African
Gestational age	38 + 3 weeks	39 + 6 weeks
Gestational issues	*Maternal:* oligohydramnios *Fetal:* bilateral hydronephrosis	*Maternal:* pre-eclampsia *Fetal:* triventricular hydrocephalus and right-sided pyelectasis
Auxological parameters at birth	BW: 0.31 SDS BL: 1.30 SDS CC: 2.11 SDS	BW: 1.19 SDS LN: 5.47 SDS CC: 2.16 SDS
Dysmorphic features	Not evident at birth	Not evident at birth
Neonatal Issues	Apnea, cyanosis and hypotonia requiring mild ventilatory support Late-onset neonatal sepsis	Apnea requiring non-invasive ventilatory support Severe cervical hypotonia, significant ligamentous laxity, poor neonatal reflexes and limited spontaneous movements
Behavioral findings	Progressive intellectual disability	Global neurodevelopmental delay
Seizures	Not present	Present and symptomatic
Cardiac anomalies	Atrial septal defect with left-to-right shunt and right ventricular enlargement	Atrial septal defect and patent ductus arteriosus
Renal anomalies	Left hydronephrosis (6 mm); two episodes of pyelonephritis	Right hydronephrosis (5 mm)
Cranial MRI abnormalities	Not performed	Areas of intracranial hemorrhage
Joint hyperlaxity	Not present	Significant ligamentous laxity
Other clinical issues	Bronchiolitis requiring non-invasive ventilatory support	Vocal cord malacia with severe obstructive dysfunction Cholestasis with dysmorphic gallbladder
*NSD1* pathogenic variant	*c.3439G* > *T,* p.(Glu1147*)	*c.2954_2955delCT,* p.(Ser985Cysfs*25)
Thyroid Aspect	Thyroid hypoplasia	Thyroid in situ
Thyroid Function at birth	TSH: 273 mIU/L fT4: 9 pmol/L	TSH: 14.67, then 16.4 mIU/L fT4: 16.6, then 18.9 pmol/L
Initial levothyroxine dose	9.5 µg/kg/day	6.5 µg/kg/day

Sotos syndrome is an overgrowth syndrome with an estimated incidence of 1 in 14,000 live births [30, 31]. It is characterized by three cardinal features: overgrowth (height and/or head circumference ≥ 2 SDS above the mean), usually of prenatal onset, learning disabilities and a distinctive facial appearance. The facial features include broad and prominent forehead with a dolichocephalic head shape, sparse frontotemporal hair, downslanting palpebral fissures, malar flushing, a long and narrow face, and a prominent chin. 15–89% of patients may exhibit behavioral disorders (most notably autism spectrum disorder), seizures, advanced bone age, cardiac anomalies, cranial MRI abnormalities, renal anomalies, joint hyperlaxity with or without pes planus, scoliosis, maternal preeclampsia, and neonatal complications [32].

Sotos syndrome is inherited in an autosomal dominant pattern. Diagnosis requires the identification of a heterozygous pathogenic variant in the *NSD1* gene or a deletion encompassing *NSD1*. More than 95% of cases have a de novo pathogenic variant [31, 32]. *NSD1* encodes a histone methyltransferase that acts as a transcriptional regulator, with both activating and repressive functions depending on cellular context. It is expressed in several tissues and organs such as the brain, kidney, skeletal muscle, spleen, thymus, and lung, though its complete function remains unclear [33, 34]. To date, no data are available in the literature regarding NSD1 expression in the thyroid.

The facial features of our female patient were not initially suggestive of classic Sotos syndrome, possibly due to ethnic variability, and became more recognizable later in life. While facial dysmorphism can support the clinical suspicion, they may not always be evident in early life. In contrast, macrocephaly is a consistent and useful diagnostic clue [35].

Although macrocephaly was already present at birth, the patient did not exhibit any other features of overgrowth initially. As shown in her growth chart (Fig. 1), overgrowth emerged around 5–6 months of age. We believe this delay may have been related to the recurrent infections and FPIES. Neurologically, only cervical hypotonia was recognized at birth; the developmental delay appeared during the follow-up, around 6 months of age. Considering that the levothyroxine treatment was started at 6 days of life and that during the endocrine follow-up TH levels remained well-controlled with appropriate dose adjustments, we attribute the neurodevelopmental delay to Sotos syndrome rather than to CH. In fact, many individuals with Sotos syndrome exhibit mild-to-severe learning disabilities, associated with behavioral issues and features of autism spectrum disorder [36].

Renal and cardiac anomalies, both present in this patient, can also be manifestations of Sotos syndrome [37]. Additionally, our patient’s neonatal complications align with features of the syndrome.

In contrast, our male patient showed overgrowth from birth, which persisted throughout follow-up (Fig. 2). He also presented with an early developmental delay, poor spontaneous motricity, and ligamentous laxity. Levothyroxine replacement therapy was initiated following the detection of altered thyroid function. However, due to inconsistency of follow-up, precise dose titration of levothyroxine was challenging. Nevertheless, despite occasional TSH rises, thyroid hormones levels always remained within normal ranges.

In the first patient, when genetic testing was performed we expected to find a gene variant responsible for CH in combination with cardiac and renal involvement, with *PAX8* as the main candidate [18]. Overgrowth features were not evident yet, but emerged over time, prompting an extended overgrowth panel (including *NSD1* and *NFIX*), which ultimately led to a definitive diagnosis. In contrast, in the second case, the onset of seizures, epileptic encephalopathy, ligamentous laxity and hypotonia guided the decision to perform a genetic test. In this case, a broad-spectrum NGS panel for encephalopathies was performed, leading to the identification of an *NSD1* mutation. In neither case was Sotos syndrome the initial suspect, highlighting the diagnostic challenges this condition presents in the first months of life. Nevertheless, both patients exhibited some common characteristics, such as macrocephaly present at birth, neonatal apnea requiring non-invasive respiratory support, cardiac abnormalities (ASD in both cases), renal anomalies (hydronephrosis), and intellectual disability. Neonatal complications, renal and cardiac anomalies and particularly behavioral abnormalities are known features of Sotos syndrome [31, 32, 38].

The *NSD1* gene variants identified in our patients, *c.3439G* > *T* p.(Glu1147*), a nonsense mutation in exon 5, and *c.2954_2955delCT* p.(Ser985Cysfs*25), a frameshift mutation also in exon 5, have both been previously reported by Saugier-Veber et al. as pathogenic variants associated with Sotos syndrome [38]. However, none of these variants were associated with CH in the literature.

Endocrine abnormalities are rarely reported in overgrowth syndromes, but may include hypothyroidism, as observed in a small number of patients affected by segmental overgrowth *PIK3CA* related disorders, Beckwith–Wiedemann syndrome and Weaver syndrome [39–41]. Specifically, limited data are currently available in literature regarding the potential association between CH and Sotos syndrome [42]. In 2005, Tatton-Brown et al. evaluated the genotype–phenotype association in 266 individuals with Sotos syndrome, reporting a case of CH due to thyroid hypoplasia with a *p.R1984X NSD1* mutation [37]. That same year, Cecconi et al. described a group of 59 patients with congenital overgrowth and reported a patient with a hypoplastic thyroid and the same *NSD1* variant [43]. In 2007, Saugier-Veber et al. described a case of Sotos syndrome with athyreosis in a cohort of 116 patients [38]. More recently, in 2021, Verma et al. reported a boy with a pathogenic novel *NSD1* variant (*c6076_6087del12*: p.(Asn2026_Thr2029del) in exon 20) and CH. Imaging findings and treatment response suggested as a possible etiology for his permanent CH either thyroid hypoplasia or a partial iodine-trapping defect [29].

Our observations further support the relationship between CH and Sotos syndrome, and we hypothesize that this relationship is unlikely to be coincidental. In both our patients, the thyroid gland was in situ (with probable hypoplasia in one case), and both required ongoing treatment beyond the age of 3 years. Notably, 4 out of 6 patients with Sotos syndrome and CH described in literature (including ours) had thyroid hypoplasia, a pattern that requires further investigation.

The small sample size and lack of functional genetic analyses limit our ability to establish a definitive causal link between *NSD1* mutations and CH. Further studies, including genome-wide association studies and functional assays investigating the role of *NSD1* in thyroid development, are needed to better determine whether CH is a coincidental association or a clinic feature of Sotos syndrome.

In the meantime, we recommend early thyroid function screening in patients with Sotos syndrome. Moreover, Sotos syndrome should be suspected in children with CH, cognitive delay, and evidence of overgrowth.

## Data Availability

Not applicable.
